# On the Regulation of the Teeth

**Published:** 1854-04

**Authors:** T. W. Evans

**Affiliations:** Paris.


### SELECTED ARTICLES.

### ARTICLE XIII.

*On the Regulation of the Teeth.* By T. W. Evans, M. D., D. D. S., of Paris.

(Continued from page 265.)

The few other innovations I have made I hope will be more successful; but they have had reference not so much to filling teeth as to regulating them, and it is to this subject that I wish, on the present occasion, to call special attention.

I need not impress upon any person in this audience, gentlemen, that irregularity of the teeth is one of the most fruitful causes of their decay. To prevent this irregularity, or where such precaution has been neglected, to remedy it, is one of the most important branches of dental science. I have, therefore, made it a particular study. Circumstances have favored this course.

Most of the persons who consult me in Paris belong to a class of society whose position renders it indispensable that whatever pertains to personal neatness and elegance should be attended to with the most scrupulous care; and in this respect no subject is of superior importance to that under consideration.

No matter what charms one may possess, whether physical or intellectual, they are all more or less neutralized by defective or irregular teeth, which at once spoil the expression of the finest countenance and destroy the effect of the most refined manners.

In a country like France, especially where the love of the elegant and the beautiful is so intense as to have become almost a worship, so important a fact could not fail to be observed and appreciated; so that now, in the refined classes of society, no one can venture to neglect it.

The "human face divine" plays too important a part in society to be neglected. Personal beauty is too intimately connected with personal grace not to require much at our hands. Nature herself, aiming always at perfection, but thwarted in a thousand ways, supplicates our assistance. She summons our *art* to the rescue. But for our response to this summons, some of her finest works—and what finer work comes from her invisible hands than a symmetrical human countenance?—would never be completed. It is our aim—and herein is the true glory of our art—to study her original design, and aid in its faithful execution.

What picture is more repulsive to the eye, and alas, what one is more common to behold than a human face, originally a type of beauty, designed as a magnificent *facade* to the "dome of thought and palace of the soul," robbed of its fair proportions, and falling prematurely to decay? And how often is this sad spectacle owing to inattention to the teeth, those delicate and beautiful organs which seem, until recent times, to have been celebrated by poets only to be "neglected of men."

The mouth, more, perhaps, than any other feature, gives character to the face. It is the natural organ of the mind. Before the thought ripens upon the tongue, if not before it glistens in the eye, it blossoms upon the lip, which, by nature, is as sensible to the emotions of the soul, as the aspen leaf to the breath of heaven. Now, that the mouth may have its full and free expression—that the organ, so to speak, may be kept in tune, its delicate ivory keys must, of course, be kept well adjusted.

One of the most striking characteristics of man, as distinguishing him from the brute, is the power of rendering his thoughts and emotions, with more or less force, in articulate language. The faculty of speech; of clothing his "thoughts that breathe" in "words that burn," is one of the noblest gifts with which heaven has endowed him. The development of this faculty is, in all countries, one of the most powerful aids of civilization. It is to this we are indebted for all the charms of oratory, and for some of the most sublime effects of music. What influence is more potent in elevating and refining the mind than the harmonious utterance of sweet sounds?

"He that hath no music in his soul,"

saith Shakspeare,

"Is fit for treason, stratagem and spoils."

And this "music in the *soul*" craves always for fit expression through the melodious instrumentality of the *voice*. Hence the education of the voice, in these great days of universal culture, has become a subject of great interest and occupies some of our best minds. Now, for the voice to have its full compass and power, that it may fitly represent the various thoughts and emotions which call for harmonious utterance, it is obvious that the sensitive organ of speech and song, the delicately constructed mouth, be as perfect as possible in all its parts. Above all things it is necessary that there should be a perfect arrangement of the teeth.

Laugh, if you please, and if your dental condition is such that you dare to, but to me an oblique tooth seems often to represent an oblique thought. Indeed, who has not remarked that such a defect will sometimes give even a sinister expression to the most benevolent idea, and rob the gentlest word of its music and its meaning.

There is a story abroad of a man whose mouth was so disfigured by the irregularity of his teeth, and the natural expression of his features was so belied by this deformity, that he regretted he could not prosecute his countenance for defamation of character.

The story is a little extravagant, I admit, but it is not without significance. I, myself, have known most estimable persons whose teeth were so mal-arranged as to give to the face an expression not only brutal, which is a common case, but almost malignant.

The question has even higher bearing. Irregular teeth often occasion irregular temper. The unfortunate creature whose dental apparatus is so badly arranged that he cannot properly digest his food becomes a dyspeptic, (often without knowing the cause,) gets nervous and irritable, ruins his constitution, and makes his friends and everybody about him, uncomfortable if not unhappy. And while in this state of mind, a man of originally good disposition loses not only his temper, but, to a considerable extent, his moral perceptions.

It is thus that the science of health is so intimately connected with the science of happiness, and that both have more to do than is commonly supposed with the science of morals.

I know, indeed, that there are said to be certain diseases which, as it were, rarify the grossness of the flesh, and make a man at once more sensible and more spiritual. But I am inclined to think, after no little observation, that no man was ever made more sensible or spiritual by the toothache; and I fancy that few men will discourse to you (at least from their own experience) of the regenerating influence of *dyspepsia.* I am skeptical also, as to the good moral or mental influence of an infected breath; there is, so to speak, an odor of ugliness about it, which is repulsive to everybody, and which can only be counteracted by an amount of personal goodness which few of us, alas, possess.

"Cleanliness," we are told, "is next to godliness," and if the proverb is true, the importance of a well-ordered mouth is so manifest that its "ivory tesselated courts" should be kept sweet and pure as a temple of worship. There are other considerations, of high moral bearing which might be presented, but I have already extended my remarks on this topic beyond what I

had intended. I trust that enough has been said, to show that irregularity of the teeth is a subject of sufficient importance to engage universal attention. And yet the world—to some extent, even the scientific world—is so much more occupied with effects than causes, with remedies than with preventives; in fine, with panaceas than with principles, that where we have one treatise on the irregularity of the teeth we have a hundred on its evil consequences.

I do not hesitate to say that nine-tenths of those who are in constant consultation with a dentist, would rarely, if ever, have had *personal* occasion for his services, if their teeth had been properly regulated in childhood. Irregular and defective teeth, now the almost universal rule would, if the proper precautions were taken, be the rare exception. Hence the pre-eminent importance of our profession; hence the family dentist is as necessary in every community as the family physician.

The eruption of the teeth of the second dentition is one of the most important stages of physical development; so that the *birth*, so to speak, of these teeth, requires the attention of the family dentist almost as much as the birth of a child requires the attention of the family physician. There is nothing like being *well-born!* Neglect in this respect, (I allude to the birth of the second teeth,) is attended, almost without exception, by serious consequences. But for this neglect, the painful extraction of teeth in mature age; tooth-ache—which has been called the "hell of diseases;"—and the necessity of wearing artificial teeth would be comparatively unknown. Hence, I do not express myself too strongly, when I say that attention to the teeth of children is not only a useful precaution but an imperative duty, and that (it cannot be repeated too often) no profession is more urgently demanded for the welfare of society than that of a family dentist. I have so constantly insisted upon these views in Paris, that my patients are now, for the most part, converted to my doctrine, and a large part of my practice is what may be called family practice. I am, in consequence, compelled to pay unusual attention to the regulation of the teeth, especially of children.

But for a long time I was seriously inconvenienced in my operations, for want of sufficiently complete apparatus. Much of that in general use I found to be often inefficient, and generally very annoying, if not injurious to the patient. I have accordingly spent much time in constructing apparatus suited to my own practice; apparatus uniting all the conditions of promptness and efficiency, and giving the least possible inconvenience to the patient. Ligatures, as generally employed, I found peculiarly open to objection; for, besides their rarely operating with sufficient certainty or steadiness, they often loosened the teeth to which they were attached for support, and almost invariably tended to lay bare the gums.

As for the other apparatus of bars, springs, caps, plates, inclined planes, &c., while there were portions of it not without merit, and some of it showing great ingenuity, it was, for the most part, so complicated or so cumbrous, that in the few cases where it accomplished the desired end, it did so only by an amount of fatigue and pain to the patient that he often found the remedy worse than the disease. These difficulties I have sought to avoid, and the success I have met with in most of my operations—some of them extremely difficult—has compensated me a thousand fold for my pains. The chief duty which remains to me is to communicate the result of my labors to you, my much esteemed colleagues, and through you to the profession at large.

The principal desiderata in apparatus for the regulation of teeth are:

1st. A firm support which shall not loosen or in any way injure the teeth to which it is attached. 2d. A steady and sufficient pressure, which can be graduated to suit particular cases, and particular stages of an operation. 3d. Great delicacy of construction that the apparatus may be as light as possible, so as neither to injure nor annoy the patient. 4th. Finally, a mechanism as simple as the case will admit of, in order to economise both labor and time.

I am aware, gentlemen, that in all these important respects great progress had been made long anterior to my departure from America. On the other hand, I presume, I am only stating the experience of every member of the profession, when I say not only that the apparatus used at that period was far from being perfect, but that there is much to be desired even now. I infer this much not only from the nature of the case, and from my own daily observation, but because in reading the valuable dental journals which come to me from America, I see that more light is called for, and less given, upon this subject than upon almost any other. Doubtless, however, many improvements have been made which have not yet been made public, and I cannot help remarking, in this connection, that the delay in publishing the valuable discoveries which are made from time to time in our profession, is deeply to be lamented, for the advance of the science depends to a great extent upon such discoveries being immediately announced. I may add, that it is possible—probable even—that some of the improvements which have occurred to me in the seclusion of my laboratory may also have occurred, in the same manner, to some of you. It is not impossible, either, that among the discoveries made here, the knowledge of which has not yet reached the Old World, there may be some far superior to my own. If so, I shall be glad to have them communicated to me, that I may introduce them into European practice.

Case I.—The first case to which I would call your attention, as illustrating the advantages of the apparatus, is that of

*Restoring an oblique upper incisor to its place.*—The first thing, of course, is to make room for the operation, if, as is generally the case, room is required. This is accomplished by bits of India rubber one-sixteenth of an inch in thickness, which are inserted between the irregular tooth and its neighbor on each side, and is prevented from sliding up to the gum by means of waxed floss silk wound tightly round the middle of the tooth. The necessary space obtained, I take a piece of fine hard-drawn wire, about the size of a common pin, and winding one end of it twice round the middle of the oblique tooth, very closely, I pass the other end along to the bicuspids or molars, to which I attach it by means of a joint—mechanism of two nicely adjusted bands or rings—forming a kind of yoke, which is slipped over two of the bicuspids or molars, and is prevented from slipping up to the gums, by means of wire clamps which hook on to their grinding surfaces. These clamps may be so constructed, when an operation requires it, as to prevent the teeth of the upper and lower jaw from coming in contact with or overlapping each other.

It will be seen at once, that if this apparatus is rightly applied, the hard drawn wire being elastic, and serving at once as lever and spring, will bring the oblong tooth round to its place in a short time. But in all cases where, from the age of the patient, or for any other cause, the tooth is too refractory to submit to so gentle a lever, I modify the apparatus as follows:

*First.*—Carefully adjusting a gold band (made of 22 carat gold, so as to be sufficiently malleable) round the centre of the tooth to be operated upon. On the front of this band, and directly across it, I solder a small gold tube, the bore of which is about the size of a common pin; through this tube, I pass a hard-drawn wire, fitting very closely, and then attach the outer end of it (which has a loop or eye for the purpose) to the yoke or skeleton cap above described, which is previously fitted to two of the molar or bicuspid teeth. In this way it will be seen, we have a more powerful lever than before, and one which is equal to any emergency. The operation being completed the tooth is kept in its place, by means of a small flattened wire, (somewhat smaller than a knitting needle,) which I pass in front of the incisors as far as the eye teeth on each side, to which it is attached by means of silk ligatures; this wire band is prevented from slipping up by means of a small frame work with two hooks, which I solder to it, and which taking hold of the cutting edge of the central incisors, afford a firm and steady support. It is obvious that the wire band thus arranged and sustained, will easily keep the restored tooth in its place. The length of time it must be worn depends upon circumstances. It should be removed as often as possible, for the purpose of cleansing.

Case II.—The next case I would submit to your judgment, is that of the

*Protrusion of the front teeth.*—I remedy this irregularity by means of a narrow elastic bar of gold, which is attached to the front of the teeth to be operated upon, by means of a kind of wire grating, constructed with hooks which take hold of the cutting edge of the central incisors, and keeps it (the bar) in its place. Having slipped this bar into the grating firmly, I pass the ends along to the two strongest molars on each side, to which it is attached, by means of short spiral springs ; the molars used for support having previously had fitted to them the yoke or skeleton cap already described. Now, to give this elastic bar, with its spiral springs, an absolutely firm and steady support, which shall have its pressure equally distributed over the mouth instead of being confined to one or two teeth, I prepare a thin gold plate of the most delicate workmanship, which is moulded carefully to the roof of the mouth, and kept in its place by attaching it to the molar teeth by the apparatus which already secures the gold bar. This plate, if nicely moulded to the irregularities of the mouth, has its pressure, as I have said, equally distributed, and renders it impossible that the teeth used for support should be loosened, since they are sustained by the whole palatine arch against which the plate presses. The elastic bar, with its spiral springs, has thus the firmest and steadiest hold possible, and can only move the teeth which it is intended to move, and these it brings easily and quickly to their place. The patient easily gets accustomed to the apparatus, because of its great delicacy of construction and finish. I should add that, as the teeth come back to their place, they will naturally force the gum inward. The plate must, therefore, be removed several times during the operation, and be modified, both in size and form ; care being taken always to have it nicely adjusted, and to keep it at least one-third of an inch from the backs of the teeth to be operated upon.

The advantages of this apparatus over the use of ligatures, as commonly applied, have already been hinted at in the course of the description. They may be summed up as follows :—1st. By avoiding the risk to which ligatures are usually liable of injuring the teeth, by slipping down to the gums. 2d. By having an absolutely firm and steady support, equally distributed over the mouth, I avoid loosening the teeth used for support, and then give the patient the least possible annoyance. 3d. By means of the gold plate fitted to the roof of the mouth, I accomplish the important desideratum of acquiring a sufficiently strong support for any power I may wish to exert upon the teeth to be operated upon, without (as I have said) running the risk of displacing any others. 4th. In fact, the power obtained by means of my elastic lever and spiral spring, with their firm, steady support, is much greater than the traction of any apparatus of ligatures, or any apparatus whatever, not thus supported, and is, therefore, easier, more prompt, and surer in its operation. To keep the front teeth in their place, after they have been restored, I first remove the plate, which had been modeled to the roof of the mouth, and then readjust it upon a new cast, attaching it by the same means as before to the molar teeth; then taking a piece of hard elastic gold wire, flattened to about one-sixteenth of an inch in width, and pass it along in front of the teeth which have been operated upon, attaching the ends to the back of the rings which have been previously readjusted to the molar teeth on each side. This elastic band, pressing gently against the teeth, easily accomplishes the purpose.

Case III.—The next case which I have thought sufficiently interesting to describe, is that when, owing to the narrowness of the jaw,

*The front teeth are forced back within the dental ridge.*—The necessary space is obtained by inserting pieces of India rubber between the teeth, in the following order:—1st. Between the central incisors, having separated them sufficiently. 2d. Then between the lateral and central incisors, having separated them sufficiently. 3d. Between the lateral incisors and the canines, retaining the India rubber in their places. The next step is to adjust the yoke or skeleton cap already described, to the two strongest molars on each side of the mouth. Next, moulding the gold plate to the roof of the mouth, in the manner mentioned in the last case, commencing pretty well back, and coming forward to within about one-fourth of an inch of the deviating teeth; this plate is attached to the molar teeth by the mechanism just alluded to—the whole forming, in fact, but one apparatus, each part of which is necessary to the other, and to the whole. Having thus secured the strongest and steadiest support, I solder one end of a gold wire (of about the size of a knitting needle) to the face of the innermost ring attached to the molar teeth, and as near as possible to the back edge. The other end of this wire, (the whole being hardened and flattened from the soldering point,) I pass along on the outside and parallel to the margin of the gum, at about one-sixteenth of an inch distance from the teeth, and slide it into a small tube, which had been previously soldered for the purpose, to the back edge of the skeleton cap, adjusted to the molars on the opposite side of the mouth. This bar is kept from slipping too far through the tube by being tapered at the end, which causes it to enter like a wedge; on the other hand, it is kept from receding from the tube by means of a small nut, or by being flattened at the end. The average width of the bar is about one-sixteenth of an inch. I curve this bar along the margin of the gum, as already described, and then attach each of the deviating teeth to it, by means of circular India rubber bands cut from a pipe or tube; these bands are first slipped over the tooth, then stretched under the bar, to be brought up on the other side and reattached to the tooth, by which means they have a firm, double hold and traction. Nothing need be added to show that these strong, elastic bands thus supported, accomplish the work of bringing forward the teeth in a manner at once simple and easy. In this case, as in the last, I retain the restored teeth in their place, by readjusting the gold plate, having first, however, added to it a kind of supplement extending up to their inner face, and carefully adapted to their posterior and irregular surface. This plate, secured as before, and with the supplemental plate resting against the restored teeth, checks their tendency to grow inwards, and thus maintains them in their proper position.

Case IV.—The next case is where the same front teeth, instead of all, being within the dental ridge, have grown irregularly—some within and others without, or what is called zig-zag. In this case, I naturally use about the same apparatus as before, with this difference: that the flattened wire or bar, is made smaller and more elastic, and can be lengthened or shortened by means of screw-nuts. The teeth which grow inwards are brought outward by means of India rubber ligatures attached to the band. I preserve these teeth in their places, by striking up a fine, thin gold plate over the backs of the six front teeth, extending down from one-fourth to three-eighths of an inch, and running over the cutting edges about one-sixteenth of an inch. This plate, made of soft ductile gold, should be carefully adapted to the inequality of the teeth, and may be generally kept in its place by the mere effect of suction, or on the principle of atmospheric pressure; but where atmospheric pressure is not sufficient, with the ordinary shape of the plate, I modify it (the plate) by making a small air-chamber in it, which I have generally found to answer the purpose. Another way of keeping the plate in its place is, to let it extend back as far as the first bicuspid, and solder a tube to it on each side, with the mouth opening against these teeth; into these tubes is inserted a piece of hickory wood which swells enough to afford a sufficient support.

Case V.—The last case I have to mention—though, if there were time, I might mention many others—is very simple, but still is not without importance. It is that of

*Bringing forward a lateral incisor.*—The skeleton cap, or yoke, is fitted to the two strongest molars; one end of the gold wire, about the size of a knitting needle, is then soldered to the back part of the yoke, and carry the other end forward to the deviating tooth, to which I attach it by means of a silk or India rubber ligature. The elasticity of the wire, which, of course, is hammered hard from the soldering point, is sufficient to bring the tooth back to its place in a short time. The apparatus for keeping this tooth in its place, is also applicable to several other cases, as the intelligent hearer will perceive. It consists simply of a thin gold plate, curved over so as to take hold of the front teeth by their cutting edge, and kept in its place by this hold, and by suction.

I have thus far spoken chiefly of the irregularity of the upper incisors; but one of the commonest cases I have to treat, is that of the

*Protrusion of the eye-teeth.*—My mode of treatment is, substantially as follows:—If the teeth are so crowded that there is hardly sufficient room for the canine to come down to its place, I bring the incisors forward in the manner already described, and in this way easily obtain the necessary space by an expansion of the dental arch. But if the space wanting is too great to be obtained in this way—in other words, if the bicuspids and incisors touch, or nearly touch each other—I extract one of the former; and then, if the tooth is so high up, that in waiting for it to come down we are likely to lose the space thus obtained, I construct an apparatus for hastening the descent of the tooth and guiding it to its place. This is applied as follows:

1st. I adjust the yoke or skeleton cap, already described, to the molar teeth, which I prevent from being loosened or displaced, by means of the gold plate moulded to the roof of the mouth. I then solder *one end* of a gold wire to the back of the skeleton cap, and having hammered the rest from the soldering point, so as to make it elastic, I curve it inwards towards the eye-tooth, against which the *other end* of the wire presses gently, operating as a spring, and aiding and directing the tooth to its place. If the protrusion be such as to require it, the wire may be crooked where it touches the eye-tooth, so as to bring it both inwards and downwards.

Another interesting case is that of the

*Protrusion of one or more of the lower front teeth.*—In this instance, if the teeth are too crowded, I extract one of the incisors, and then bring the others together by means of India rubber rings slipped over the teeth on each side of the space thus obtained. I then form a cap, by bending a piece of flattened gold wire, or plate, over the cutting edge of the irregular tooth, extending down almost to the gum on each side, and thus embracing the whole crown. The ends of this cap where they approach the gum are turned up so as to form small eyelets or hooks. The cap is kept in its place by means of a silk ligature wound tightly round it, which silk is kept from slipping down to the gums by the hooks or eyelets. Having adjusted this simple mechanism, and fitted a skeleton cap to the molars used for support, I run a spiral spring from the irregular tooth to the back of this cap, attaching it at each end by little hooks. The operation of this spiral spring brings the tooth back to its place steadily and promptly. If the spring interferes too much with the tongue, or prevents articulation, I pass a gold wire through it, curved inward to the form of the gum, and soldered at one end upon the skeleton cap. The spiral spring thus curved leaves the tongue entirely free, while it operates as perfectly upon the wire as if it were run directly from the tooth to the skeleton cap.

I have thus, gentlemen, given you, as clearly as is possible for me to do in a public lecture, some of the results of my labors in the way of regulating teeth. If I have failed to make myself perfectly clear, I shall be happy to make any explanations which may be required of me. For, in my view, gentlemen, ours is a science far too noble in its character, and involving far too many general interests, to be hampered with any monopolies. A patent for a particular mode of regulating teeth, seems to me as absurd as a patent for a particular mode of setting a bone or curing a fever. There is something almost sacrilegious in the very idea. Everything we discover for the benefit of human kind, should be known and read of all men. It should be published in the streets, and proclaimed from the house-tops, that thus we may humbly imitate that generous Providence, which sends its rain alike upon the just and the unjust, and scatters its light, broad-cast, over the whole face of the earth.

